# Construction of a chromosome-level Japanese stickleback species genome using ultra-dense linkage analysis with single-cell sperm sequencing

**DOI:** 10.1093/nargab/lqac026

**Published:** 2022-03-31

**Authors:** Kazutoshi Yoshitake, Asano Ishikawa, Ryo Yonezawa, Shigeharu Kinoshita, Jun Kitano, Shuichi Asakawa

**Affiliations:** Laboratory of Aquatic Molecular Biology and Biotechnology, Graduate School of Agricultural and Life Sciences, The University of Tokyo, 1-1-1 Yayoi, Bunkyo, Tokyo, Japan; Ecological Genetics Laboratory, National Institute of Genetics, Yata 1111, Mishima, Shizuoka 411-8540, Japan; Department of Integrated Biosciences, Graduate School of Frontier Sciences, The, University of Tokyo, 5-1-5 Kashiwanoha, Kashiwa, Chiba 277-8561, Japan; Laboratory of Aquatic Molecular Biology and Biotechnology, Graduate School of Agricultural and Life Sciences, The University of Tokyo, 1-1-1 Yayoi, Bunkyo, Tokyo, Japan; Laboratory of Aquatic Molecular Biology and Biotechnology, Graduate School of Agricultural and Life Sciences, The University of Tokyo, 1-1-1 Yayoi, Bunkyo, Tokyo, Japan; Ecological Genetics Laboratory, National Institute of Genetics, Yata 1111, Mishima, Shizuoka 411-8540, Japan; Laboratory of Aquatic Molecular Biology and Biotechnology, Graduate School of Agricultural and Life Sciences, The University of Tokyo, 1-1-1 Yayoi, Bunkyo, Tokyo, Japan

## Abstract

It is still difficult to construct the genomes of higher organisms as their genome sequences must be extended to the length of the chromosome by linkage analysis. In this study, we attempted to provide an innovative alternative to conventional linkage analysis by devising a method to genotype sperm using 10× Genomics single-cell genome sequencing libraries to generate a linkage map without interbreeding individuals. A genome was assembled using sperm from the Japanese stickleback *Gasterosteus nipponicus*, with single-cell genotyping yielding 1 864 430 very dense hetero-SNPs and an average coverage per sperm cell of 0.13×. In total, 1665 sperm were used, which is an order of magnitude higher than the number of recombinations used for conventional linkage analysis. We then improved the linkage analysis tool scaffold extender with low depth linkage analysis (SELDLA) to analyze the data according to the characteristics of the single-cell genotyping data. Finally, we were able to determine the chromosomal location (97.1%) and orientation (64.4%) of the contigs in the 456 Mb genome of *G. nipponicus*, sequenced using nanopores. This method promises to be a useful tool for determining the genomes of non-model organisms for which breeding systems have not yet been established by linkage analysis.

## INTRODUCTION

The development of next-generation sequencing and associated technologies has improved the ease of genome decoding and has enabled the construction of bacterial genomes of several million bases using nanopores and PacBio (1,2). However, higher eukaryotes often have genomes that are >100 times larger than those of bacteria and contain large repetitive sequences, such as centromeres, telomeres and rRNA clusters, making them difficult to decipher. During genome assembly, linkage analysis or Hi-C are used to extend the scaffolds up to the lengths of chromosomes after short reads have been assembled and contigs have been scaffolded (3–5). Although methods have been established for constructing chromosome-level genomes, the steps to get from scaffolds to chromosomes remain challenging.

Of the 18 568 eukaryotic genomes in the NCBI genome database (https://www.ncbi.nlm.nih.gov/genome/browse#!/eukaryotes/) (24 July 2021), only 15% have been extended to chromosomes, whereas 62% are scaffolds and 23% are contigs. Consequently, the genomes of many organisms have not yet been constructed at the chromosome level, potentially owing to unresolved difficulties when conducting linkage analysis and Hi-C. For example, in linkage analysis, it is necessary to prepare the DNA of both the parents and offspring to detect recombination in the parental germ line cells; however, this is often difficult in species with undetermined genomes because their breeding systems have not been established. Recently developed Hi-C technology, which utilizes the frequency of interactions along DNA molecules in the cell nucleus, can only be used to analyze a single individual and can detect genomic interactions with a resolution of 1 kb (6). However, there are several disadvantages of Hi-C. Depending on the software used, Hi-C analysis can produce different genome structures (7) and can link different chromosomes together (8).

**Figure 1.**
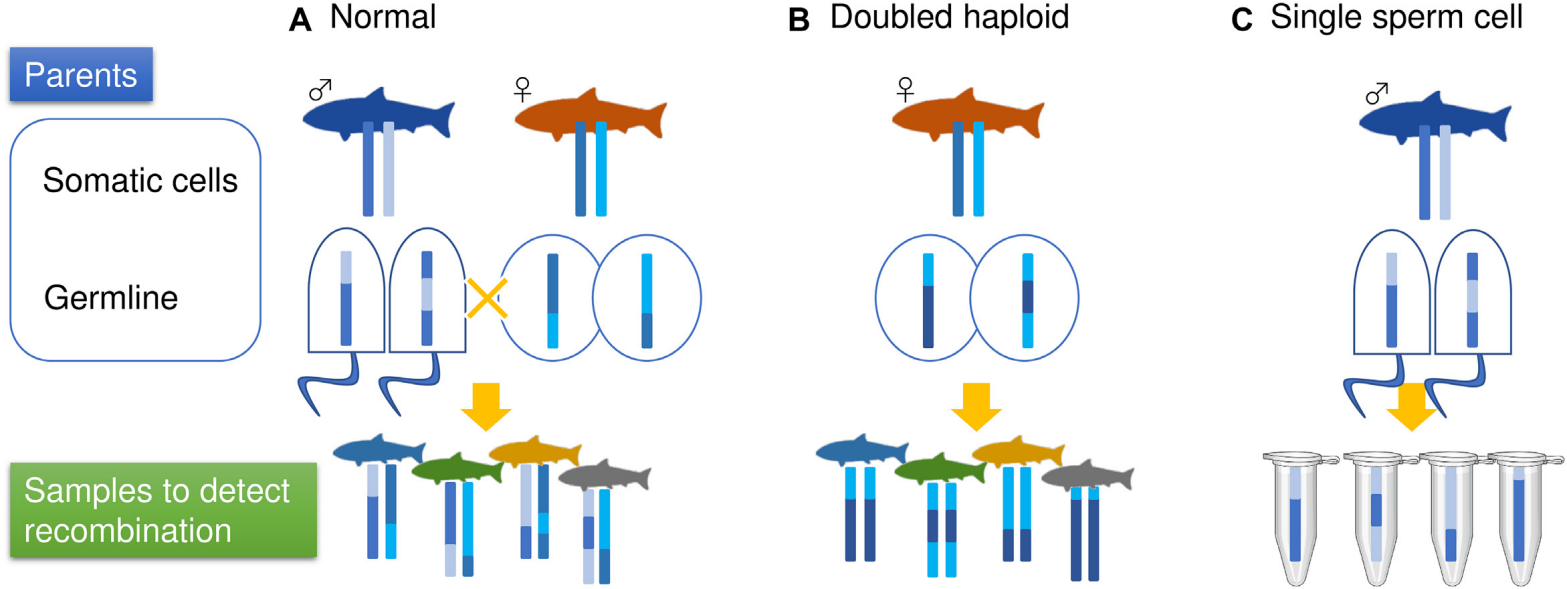
Comparison of linkage analysis methods. (**A**) Normal linkage analysis. The father and mother each have a diploid genome, whereas the children inherit two of the parent's four total haplotypes. The genome of hetero-diploid children will be used to detect recombination. (**B**) Double haploid linkage analysis. Recombination is detected in children with a homozygous diploid genome produced by artificial parthenogenesis from the mother’s oocyte. (**C**) Single sperm cell linkage analysis. Recombination is detected in the haploid single sperm after whole-genome amplification.

Previously, we developed chromosome-level genome construction methods based on linkage analysis using female monozygotic individuals (doubled haploid) (9), as well as hybrids (3) (Figure 1). Coverage of 30× or more is typically required to genotype a diploid genome, whereas a haploid genome only requires 1× coverage. Accordingly, we were able to drastically improve marker resolution and create a very accurate linkage map of some species that can be bred as doubled-haploid individuals or hybrids using the 1× coverage strategy. In this study, we further developed this doubled haploid and hybrid system and incorporated recently developed single-cell technology to genotype single sperm cells. Each cell was assigned a different barcode sequence in a 10× Genomics library conditioning system and was read immediately using an Illumina sequencer (Figure 1). Using this system, it is possible to easily sequence even 10 000 cells by increasing the number of reads and to directly detect recombination in parental germ line cells without breeding their offspring, since single sperm cells can be genotyped. Furthermore, it is possible to efficiently genotype haploid sperm and create a dense linkage map from the 1× and smaller data volume cultivated using our doubled haploid and hybrid system.

Although single sperm cell genotyping has been reported in species such as humans and *Daphnia* (10,11), the number of cells analyzed is as low as 104. Using the 10× Genomics single-cell system, it is realistically possible to analyze 10 000 or more sperm. If 10 000 individual human sperm can be analyzed, recombination could be detected at approximately once every 10 kb since the genetic distance is approximately 1 cM = 1000 kb, which will result in one recombination in every 100 individuals per 1000 kb (12). Therefore, if recombination occurs in a scaffold, its position and orientation in the final chromosome can be completely determined. In the past, scaffolds typically had to be of Mb-size for linkage analysis owing to the small number of markers and individuals to be used. For example, conventional linkage analysis can only use up to 100 individuals and 10 000 markers. We developed SELDLA software as a tool for ultra-high-density linkage analysis in doubled-haploid and crossbreeding species and improved this software to analyze single-cell sperm systems. In this study, single-cell whole-genome amplification was performed on *Gasterosteus nipponicus* sperm using 10× single-cell genome sequencing libraries. *G. nipponicus* has been investigated in the context of speciation and freshwater fish colonization (13,14) and is closely related to the extensively studied three-spined stickleback (*Gasterosteus aculeatus*) (15,16), which has a relatively small 468 Mb genome compared to that of other vertebrates. *G. nipponicus* has recently been used as an important fish for evolutionary studies, but its genome assembly has not to be determined. Determining the genome of *G. nipponicus* will provide profound insights into the evolution of three-spined stickleback. Although the 10× Genomics single-cell sequencing of pollen for linkage analysis has been reported (8), to our knowledge, this study is the first to conduct 10x Genomics single-cell sequencing and linkage analysis using sperm. In addition, we improved our SELDLA software to enable easy linkage analysis.

## MATERIALS AND METHODS

### Sperm collection from *G. nipponicus* and DNA extraction from the liver, fin and pectoral muscles

*Gasterosteus nipponicus* fish were donated by a local fisherman from Akkeshi Bay (Hokkaido, Japan) in May 2019. After collection, the fish were maintained in 50% seawater at 16°C under long-day conditions (16L8D) for 6 months. After the fish had been sacrificed with an overdose of buffered MS222 (500 mg l^−1^), the male testes were excised and disrupted in 200 μl of Leibovitz’s L-15 Medium with 10% fetal bovine serum (FBS) to release sperm, which were dispersed by pipetting. The cell mass was removed using a 100 μm cell strainer (Falcon), and the cell suspension was diluted to 1/100 before the cells were counted using an automated cell counter (Millipore). Sperm cell suspensions were then centrifuged at 500 × *g* and 4°C for 5 min and the supernatant was removed, resuspended in 400 μl of cryopreservation solution (FBS: N,N-dimethylformamide = 9: 1) in a Cryotube, chilled for 20 min in a 15 ml tube spiked with dry ice, and stored in liquid nitrogen. All other body parts were preserved in 100% ethanol before DNA was extracted from the liver, fin, and pectoral fin muscles using a DNeasy Blood & Tissue Kit (QIAGEN). All animal experiments were approved by the Institutional Animal Care and Use Committee of the National Institute of Genetics (31–16).

### Nanopore sequencing

Short fragments of <7 kb were removed using short read eliminator XS (SRE; Circulomics) according to the manufacturer’s protocol but with a 50°C elution temperature using 50 μl of Buffer EB. The amount of DNA after SRE elution was measured using a Qubit 2.0 (Thermo Fisher Scientific) with a Qubit dsDNA BR Assay Kit (Thermo Fisher Scientific). The total yield was 5.5 μg. To determine the length of DNA fragments after SRE, we used undiluted solution after SRE treatment together with pre-SRE gDNA diluted 5-fold with the TapeStation 2200 (Agilent Technologies) and Genomic DNA ScreenTape (Agilent Technologies) according to the manufacturer's protocol.

The starting amount of DNA was adjusted to approximately 1.2 μg, and a library was prepared using a Ligation Sequencing Kit SQK-LSK109 (Nanopore) according to the manufacturer’s protocol. DNA concentration was measured using a Qubit 2.0 with Qubit dsDNA BR Assay Kits. Next, a Flow Cell R9.4.1 (Nanopore) was washed twice, and sequencing was performed a total of three times, with the following number of active pores, initial bias voltage, and sequencing times: first run, 1432, −180 mV, 13 h 57 m; second run, 1136, −190 mV, 19 h 48 m; third run, 901, −210 mV, 72 h 8 m. The flow cell was washed using EXP-WSH003, with the reaction time of the WashMix changed from 30 min to 2 h and the protocol followed for the rest of the process. Bases were called using MinION software v19.12.5.

### Sperm single-cell analysis

Frozen sperm were sent to GENEWIZ® for 10× Genomics single-cell copy number variation (CNV) library preparation and sequencing. The cells were prepared according to a previous single sperm cell analysis study (17). A single-cell library was constructed according to the manufacturer’s protocol. Sequencing was performed in three lanes using a HiSeq 4000 (Illumina) with paired-end sequencing of 150 bp.

### Creating contigs

Nanopore-sequenced data were assembled using Flye (v2.7) (18) with the following options: ‘–nano-raw –genome-size 400M –threads 12’. The assembled contigs were mapped with the Nanopore sequencing data using minimum (v0.2) (19) and polished with Racon (v0.5.0) (20). In total, 500 000 000 reads (311× higher than the genome) were extracted from the single-cell Illumina data. After 16 bp on the forward side corresponding to the 10× Genomics cell barcode had been removed, the Racon-polished genome was mapped with the Illumina sequencing data using BWA MEM (v0.7.15) (21) and was corrected using Pilon (v1.23) (22).

### Single-nucleotide polymorphism (SNP) extraction

Single-cell CNV analysis was performed using Cell Ranger (ver 3.0.2; 10× Genomics) using the Pilon polished genome as a reference sequence. Since Cell Ranger does not consider contigs <1 Mb long, we created and analyzed pseudo-chromosomes with short contigs connected by N × 10 000. Cell Ranger CNV analysis produced several tags containing multiple sperm; therefore, we removed tags that were judged to be diploids using the ploidy_confidence score. Vartrix (v1.1.14; 10× Genomics) was used to call SNPs in single cells after Bcftool mpileup (v1.10.2) (23) had been used to create a bulk vcf file of all SNPs from 500 000 000 reads from single cells. The SNP matrix file obtained using vartrix was converted to a cell-separated vcf format. The script is available as linkage-analysis∼single-cell_CellRanger-VarTrix in Portable Pipeline (https://github.com/c2997108/ OpenPortablePipeline), which has a graphical user interface for user-friendly operation and executes jobs from Windows/Mac to a remote Linux server.

### Improvement of SELDLA

We have improved SELDLA to handle single cell linkage analysis data at the step of imputation. In the case of crossbred data, the coverage was approximately 1×, and the missing markers were rare, and thus, there were few problems in referring only to the nearest valid marker of the missing marker. However, in the case of single-cell data, the coverage was approximately 0.1×, and there were many missing values, and therefore, we found that referring only to the nearest neighbor markers would have a large impact if those markers were wrong. Therefore, we changed the method to use three valid markers in the neighborhood for imputation. The number of nearby markers to be referenced can be changed as an option.

### Genome elongation using linkage analysis

Called cell-separated vcf files were analyzed using SELDLA (v2.1.2) (3) to extend contigs to chromosomes with the following options: ‘–mode = haploid -p 0.03 -b 0.03 –cs 2 –nl 0.9 –NonZeroSampleRate = 0.05 –NonZeroPhaseRate = 0.1 -r 4000 –RateOfNotNASNP = 0.001 –RateOfNotNALD = 0.01 –ldseqnum 3 –noNewVcf’. Cells with apparent recombination between >5% of markers were deemed not to be single cells and were removed. Then, SELDLA was run again using the same parameters using filtered single cells as input. This analysis pipeline is available as the linkage-analysis∼SELDLA script in Portable Pipeline. Constructed genomes were evaluated by BUSCO (v3.0.1) (24) with the database of ‘eukaryota_odb9’.

### Centromere search

To search for centromeres, the centromere sequence (25) of *G. aculeatus* was searched as a query using blastn (v2.6.0+) (26) with default parameters. Hit locations were considered centromeres.

### Comparative genomics

Minimap2 (v2.13) (27) was used to compare homologous regions between *G. aculeatus* (28) and *G. nipponicus*. Regions with an alignment of >1000 bp were visualized as dot plots. The script is available as post-assemble∼dotplot-by-minimap2 in Portable Pipeline.

## RESULTS

### Single-cell library construction

Sperm were collected from a *G. nipponicus* individual captured at Akkeshi Bay. Single-cell libraries were constructed with 10x Genomics Chromium using approximately 300 000 sperm and were sequenced using HiSeq. In total 1 276 740 950 × 2 paired-end reads of 383 Gb were sequenced (Table 1).

**Table 1.** 10x Genomics single-cell genome (CNV) and Nanopore sequencing results

	10× single-cell	Nanopore
**Tissue**	Sperm	Fin and pectoral fin muscle
**Number of sequenced reads**	1 276 740 950 × 2	2 163 108
**Sequenced bases**	383 Gb	8.1 Gb
**Sequencing depth per genome**	840×	17.8×
**Number of sequenced cells**	2286	-
**Number of sequenced haplotype-candidate cells**	1738	-
**Number of sequenced haplotype cells**	1665	-
**Number of heterozygous SNPs**	1 864 430	-
**Average coverage per cell**	0.13×	-

### Nanopore sequencing

DNA was extracted from the fin and pectoral fin muscles of the individual from which the sperm was collected, and the size of the resulting DNA fragments was checked using a TapeStation (Supplementary Figure S1A). Since 7–60 kb fragments accounted for only 57.95% of the total reads, short reads were removed using SRE (Supplementary Figure S1B), enriching the 7–60 kb long fragments to 76.54% (Supplementary Figure S1C). In total, 2 163 108 reads were obtained by nanopore sequencing, with a total base count of 8.1 Gb (17.8×) and a read N50 of 5.87 kb. N50 is defined as the sequence length of the shortest contig at 50% of the total genome length. The sequenced reads were assembled using Flye software to obtain 3895 contigs with an N50 of 0.563 Mb and a BUSCO score of 81.2% (Table 2). The assembled contigs were polished using nanopore and single-cell HiSeq reads to produce a post-polish genome with an elevated BUSCO score of 96.7% (Table 2), confirming that the sequencing errors could be corrected.

**Table 2.** Statistical analysis of the constructed genome

	Number of contigs	N50 [Mb]	Total [Mb]	BUSCO [%]	Placed bases in chromosomes [%]	Oriented bases in chromosomes [%]
**Assembled**	3895	0.563	455	81.2	-	-
**Polished**	3668	0.565	456	96.7	-	-
**After linkage analysis**	3095	12.71	456	97.1	97.1	64.4

### Genotyping single sperm cells and filtering tags for multiple sperm

The sequenced data were analyzed with 10× Genomics single-cell genome sequencing libraries using Cell Ranger software to detect CNVs in each cell, with the polished genome as a reference genome. Cell Ranger detected 2286 cells (Table 1), among which 1738 haplotype-candidate cells remained after cells that appeared to have been mixed with multiple spermatozoa had been removed. In total, 1 864 430 heterozygous SNPs were detected in these cells, meaning that there was one SNP per 245 bases in the genome of 456 Mb. The average read coverage per cell was 0.13. This is comparable to the past research with 10× CNV kits, specifically 369 cells and 0.1× coverage per cell (8).

### Genome construction by linkage analysis of single sperm cells

**Figure 2.**
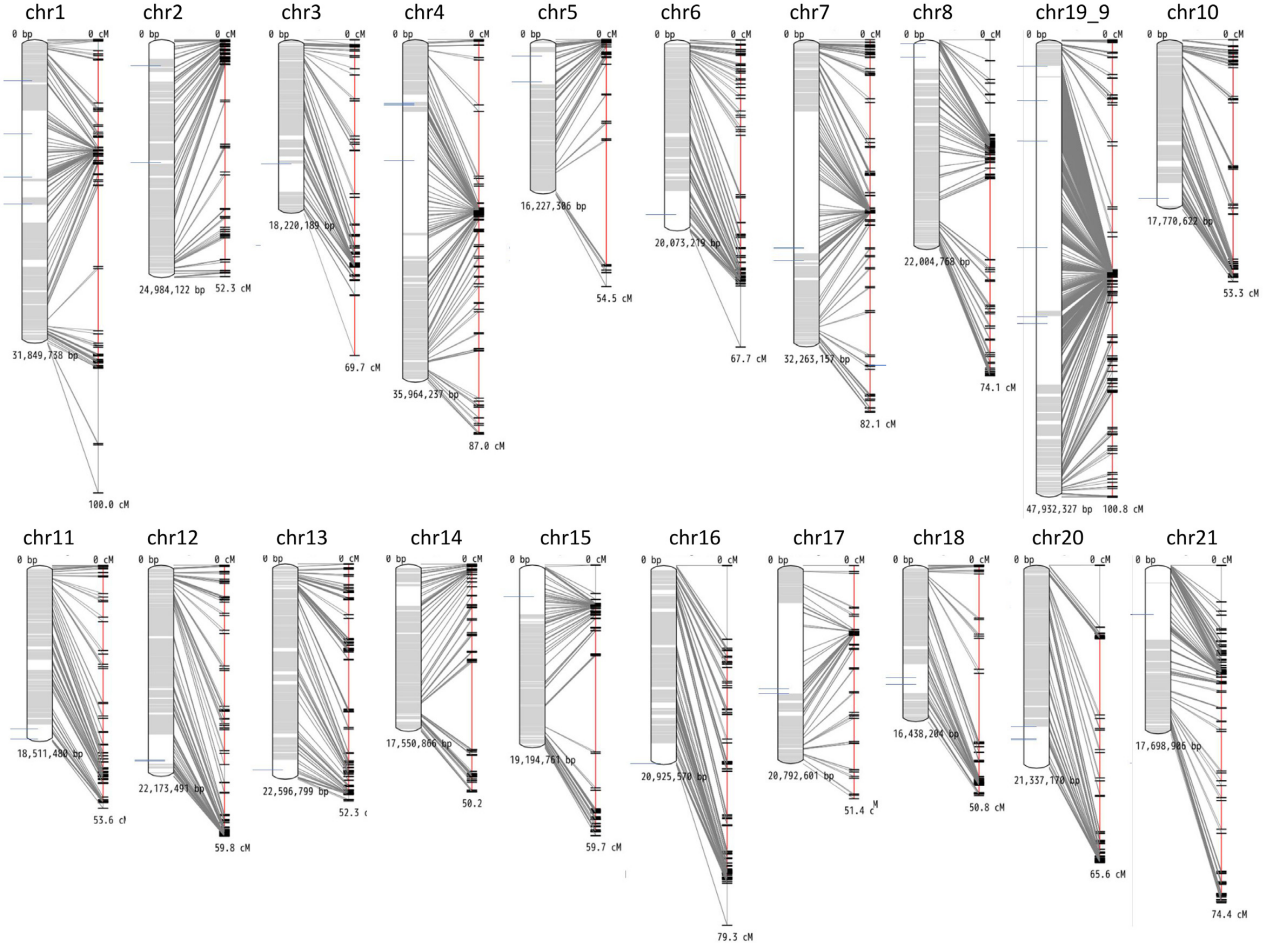
Physical map and linkage map of the genome constructed using single sperm cell linkage analysis. Correspondence between physical (left) and genetic (right) distances is shown for scaffolds elongated to >10 Mb. Areas where contig orientation on the chromosomes could be determined are shown in gray. Areas where contig orientation could not be determined are shown in white. Lines indicating correspondence between the physical and linkage maps are based on the positions of both ends of the contigs. The blue line shows the centromeric repeat sequence (accession ID: KT321856.1) of the *Gasterosteus aculeatus* hit. All chromosomes except chr14 had hits for the centromeric repeat sequence in white loci where contig orientation could not be determined.

The extracted SNPs were inputted into SELDLA for linkage analysis. The first SELDLA run identified several cells that appeared to have an extremely high number of breakpoints. These cells were thought to be a mixture of multiple spermatozoa on a single tag or a mixture of immature diploid spermatozoa that had not yet completed their second meiosis. Cells that appeared to share >5% of breakpoints between markers were considered not haploid cells and were removed, leaving a total of 1665 cells for the second SELDLA analysis run that resulted in 20 scaffolds of >10 Mb (Figure 2). The SELDLA-extended scaffold had an N50 of 12.71 Mb (Table 2), and 97.1% of contigs could be placed in linkage groups, whereas the orientation of 64.4% of contigs could also be determined. This is the first determined genome of *G. nipponicus*. In most chromosomes, the centromeric repeat homologs were plotted in a single region for which the orientation could not be determined (Figure 2), but in chr1, chr2, chr4, chr7 and chr19_9, the centromeric repeat homologs were scattered in different genetic position. This could be due to errors in the assembly or the linkage map.

### Comparison of the constructed genome with a closely related species

**Figure 3.**
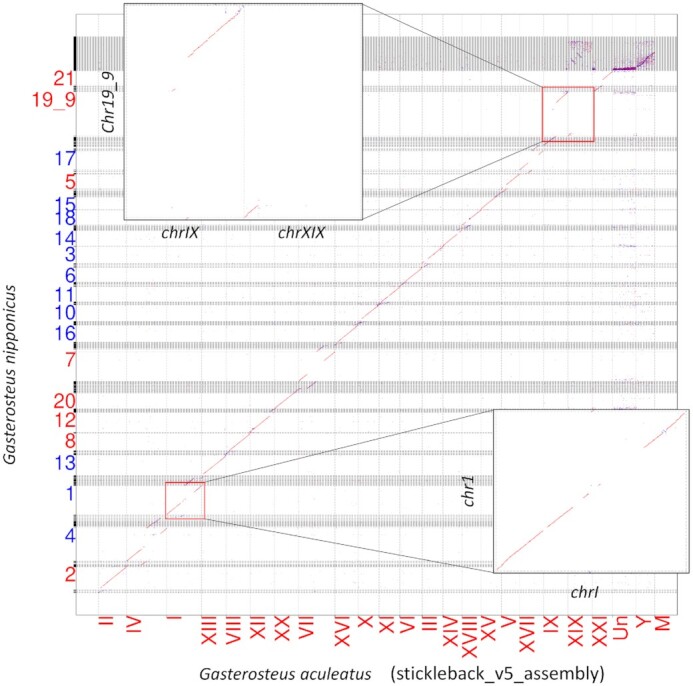
Whole genome dot plot analysis of *Gasterosteus nipponicus* and *Gasterosteus aculeatus*. Dot plot comparing the scaffold extender with low depth linkage analysis (SELDLA)-elongated *G. nipponicus* chromosome (*y*-axis) and the published *G. aculeatus* reference sequence (*x*-axis). Red lines in the dot plot indicate homology in the forward direction. Blue lines indicate homology in the reverse direction. Only chromosome numbers are labeled, and labels for small contigs that were not incorporated into the chromosome are omitted. Red indicates that the chromosomes are arranged in the forward direction and blue indicates the reverse direction.

Next, we compared the *G. nipponicus* genome generated in this study with the published genome of a closely related species, *G. aculeatus* (Figure 3 and Supplementary Figure S2). Since no recombination had occurred around the centromere, the contigs near the centromere could be located but their orientation or order within the non-recombinant locus could not be determined in our *G. nipponicus* assembly. These unoriented contigs were outputted as chromosome-associated contigs using different IDs for the chromosomes and denoted by N in the chromosomes, and thus, their homology with *G. aculeatus* was not calculated, leaving several blank spaces in the dot plot of the centromere of *G. nipponicus*. However, the homologous regions between *G. nipponicus* and *G. aculeatus* chromosomes were clearly aligned on the diagonal (Figure 3). No major genomic rearrangements between the *G. nipponicus* and *G. aculeatus* genomes were observed in this study, except for with the sex chromosome chr19_9. Chromosomes 19 and 9 of *G. aculeatus* have previously been reported to be linked to a neo-sex chromosome in *G. nipponicus* in which chromosomes 9 and 19 are fused together (29). The 64.4% of contigs were determined the orientation (Table 2) and 97% of oriented contigs were aligned in the same direction between *G. nipponicus* and *G. aculeatus* (Figure 3), which indicate the accuracy of our method.

### Effect of cell number

**Figure 4.**
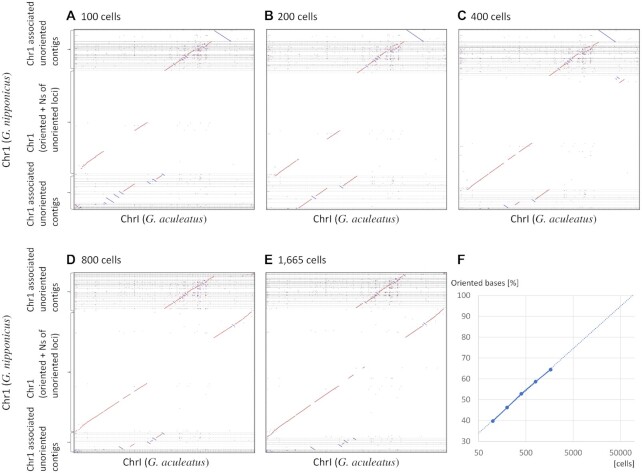
Increasing the number of cells increases the percentage of contigs that could be oriented. Dot plots comparing chr1 of *Gasterosteus aculeatus* (*x*-axis), homologous chr1, and genome contigs constructed using 100 (**A**), 200 (**B**), 400 (**C**), 800 (**D**) and 1665 (**E**) cells (*y*-axis). Chr1 of *Gasterosteus nipponicus* was filled with Ns where the orientation could not be determined. Dot plots show gaps in *G. nipponicus*, corresponding to the loci filled with Ns. (F) Relationship between the number of cells used and the number of bases in the contigs for which the orientation could be determined.

Although it is difficult to breed 100 siblings for traditional linkage analysis, the number of cells can easily be increased in single-cell experiments. In this study, we used 1665 cells and investigated how the linkage analysis results would be affected by reducing the number of sperm cells to 100, 200, 400 or 800. The constructed genomes were compared with the genome of *G. aculeatus* (Figure 4A–E, Supplementary Figures S3–S6 and Figure 3). When only 100 cells were used, the contigs that could not be oriented were located mostly on the chromosome; however, the number of contigs that could be oriented around the centromere gradually increased by approximately 6% as the number of cells increased two-fold (Figure 4F). Although we could not determine the orientation of contigs around the centromere, even with 1665 cells (Figures 2 and 4A–E), we predicted that we could determine the orientation of >90% when using 50 000 cells.

## DISCUSSION

In this study, we established an innovative genome construction method for genotyping sperm using 10× Genomics single-cell genome sequencing libraries and by generating linkage maps without interbreeding. The detected SNP markers were very dense (1 864 430), and we were able to analyze 1665 sperm, which is an order higher than that which can be analyzed using conventional linkage analysis. Theoretically, >97.1% of contigs could be placed, and 64.4% could be oriented on the chromosomes, and the genome was constructed with a high accuracy with the BUSCO score of 97.1% (Table 2). Together, our findings indicate that linkage analysis using single sperm cells is very versatile and could become a standard genome analysis method in the future. However, our results detected some misassemblies including the centromeric repeats (Figure 2). Our method was able to incorporate even small contigs into the linkage group by using very dense markers. As a result, our *G. nipponicus* genome contains 1695 centromeric repeats, whereas *G. aculeatus* contains only 477 centromeric repeats. More contigs can be placed on the chromosomes of *G. nipponicus*, but some of them may have been misplaced. These misassemblies can be verified with long reads, Bionano, Hi-C and another linkage analysis in the future.

The presence of non-recombining regions in sex chromosomes is an issue when genome assembly is based on linkage analysis. For example, in several species, including mammals, X and Y chromosomes can recombine only within limited pseudo-autosomal regions (30), meaning that a linkage map cannot be drawn for the non-recombining region using sperm. However, some species of fish have homomorphic sex chromosomes. For example, X and Y chromosomes differ by only a few genes in medaka (31) and only one base in pufferfish (32), with recombination occurring between almost the entire X and Y chromosomes in these species. Therefore, it could be possible to apply our method to sex chromosomes of such species with homomorphic sex chromosomes.

We confirmed that the recombination rate around the centromere was very low in *G. nipponicus*, as in the three-spined stickleback (33), and therefore, >30% of contigs could not be oriented on the chromosomes because no recombination occurred in the 1665 cells. Previous studies have also shown that recombination near the centromere in males is highly suppressed (34) in pufferfish (35) and dogs (36). However, recombination near the centromere is not suppressed in humans (12) and *Arabidopsis* (37), whereas recombination across the entire chromosome is more likely in males more in grasshoppers (38), and there is no sex-specific difference in recombination suppression in most birds and maize (34). Although it was difficult to determine the assembly of centromeres in the stickleback, our method would be applicable to other species that have more recombination events near centromeres in sperm (34). Even though the recombination rate was low, we were able to increase the percentage of oriented contigs by approximately 6% by doubling the number of sperm. Therefore, a further increase in the number of sperm could improve the assembly as far as recombination occurs to some extent even at low frequencies. In this study, it was shown that it is possible to construct a genome by using a dense linkage map even with a relatively short contig N50 of 0.565 Mb. If longer contigs could be used, more contig orientations could be determined.

In this study, we established a single-cell linkage analysis method using sperm, which does not require any laboratory breeding and allows for accurate linkage analysis, and applied it for the chromosomal-level genome assembly of a Japanese stickleback species with 456 Mb. Using a high density of >1 million SNP linkage markers, we were able to determine the contig location of 97.1% of the genome. In recent years, genome analysis methods that do not rely on linkage analysis, such as Hi-C, have emerged to obtain relationships between DNA sequences with longer distances than the DNA fragment length obtained by DNA extraction. However, since the genome structure constructed using Hi-C is known to create chromosomal chimeras (8), it is very important to compare the genome structure with the results of linkage analysis. Sequencer throughput and the technology for preparing single-cell libraries are rapidly evolving; therefore, it might be possible to perform linkage analysis using >10 000 single sperm in the future. The increase in the number of cells used will dramatically improve the accuracy of single cell linkage analysis. This method is very useful for determining the genomes of non-model organisms for which breeding systems have not yet been established.

## DATA AVAILABILITY

All sequencing data are available in the DNA Data Bank of Japan (DDBJ) under the accession number ‘PRJDB9841’. *Gasterosteus nipponicus* genome sequences are available in DDBJ under the accession number ‘BMAF01000001-BMAF01003095.’

## CODE AVAILABILITY

SELDLA software is available at https://github.com/c2997108/SELDLA. Portable Pipeline is available at https://github.com/c2997108/OpenPortablePipeline.

## Supplementary Material

lqac026_Supplemental_File
