# Supplementary material for: Construction of a chromosome-level Japanese stickleback species genome using ultra-dense linkage analysis with single-cell sperm sequencing
Source: NAR Genom Bioinform. 2022 Mar 31;4(2):lqac026. doi: 10.1093/nargab/lqac026 (PMC8969643; doi:10.1093/nargab/lqac026)
Supplement: lqac026_Supplemental_File [file lqac026_supplemental_file.pptx]

## Slide 1
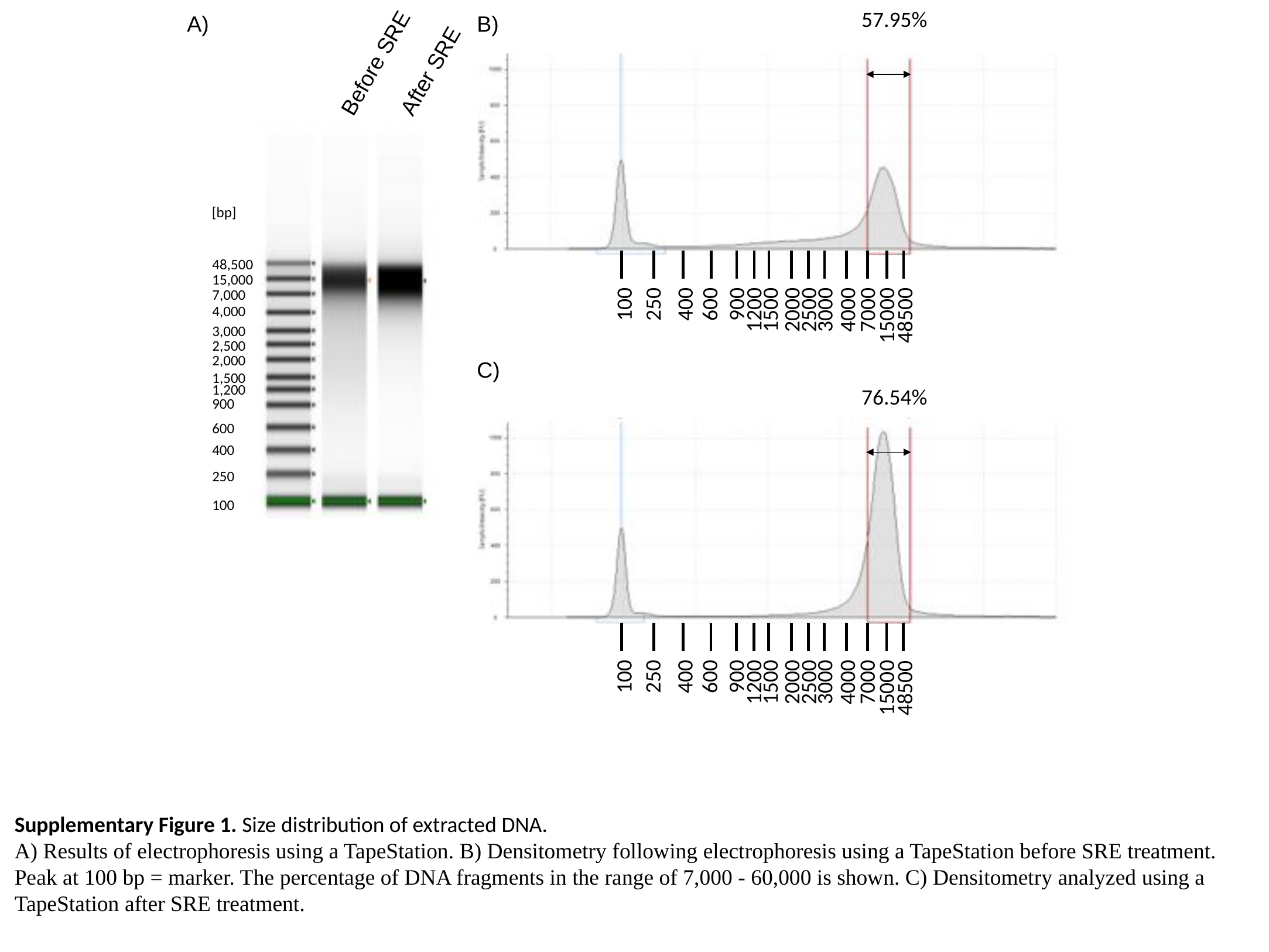

57.95%
A)
B)
Before SRE
After SRE
[bp]
48,500
15,000
7,000
100
250
400
600
900
1200
1500
2000
2500
3000
4000
7000
4,000
15000
48500
3,000
2,500
2,000
C)
1,500
1,200
76.54%
900
600
400
250
100
100
250
400
600
900
1200
1500
2000
2500
3000
4000
7000
15000
48500
Supplementary Figure 1. Size distribution of extracted DNA.
A) Results of electrophoresis using a TapeStation. B) Densitometry following electrophoresis using a TapeStation before SRE treatment. Peak at 100 bp = marker. The percentage of DNA fragments in the range of 7,000 - 60,000 is shown. C) Densitometry analyzed using a TapeStation after SRE treatment.

## Slide 2
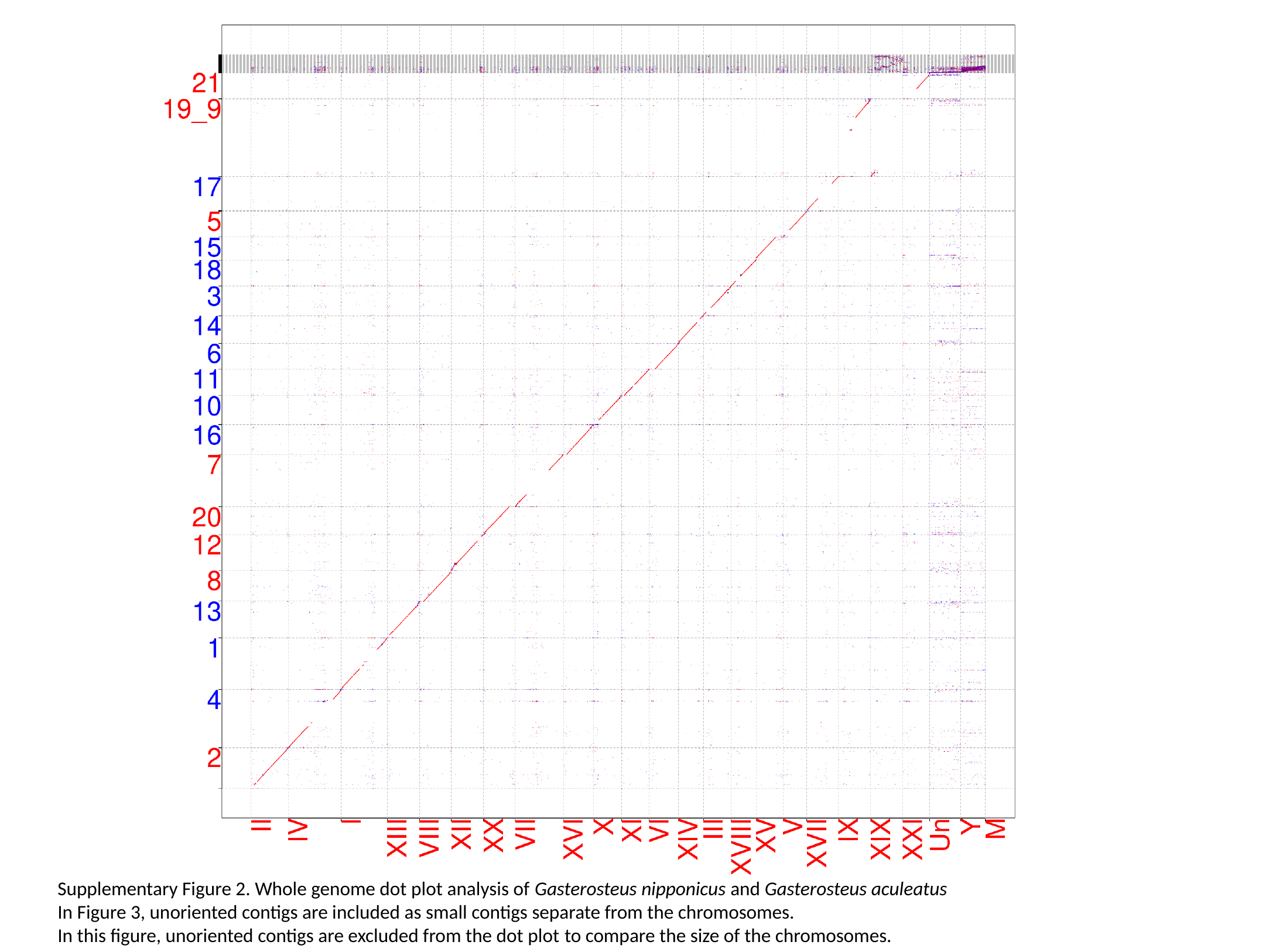

Supplementary Figure 2. Whole genome dot plot analysis of Gasterosteus nipponicus and Gasterosteus aculeatus
In Figure 3, unoriented contigs are included as small contigs separate from the chromosomes.
In this figure, unoriented contigs are excluded from the dot plot to compare the size of the chromosomes.

## Slide 3
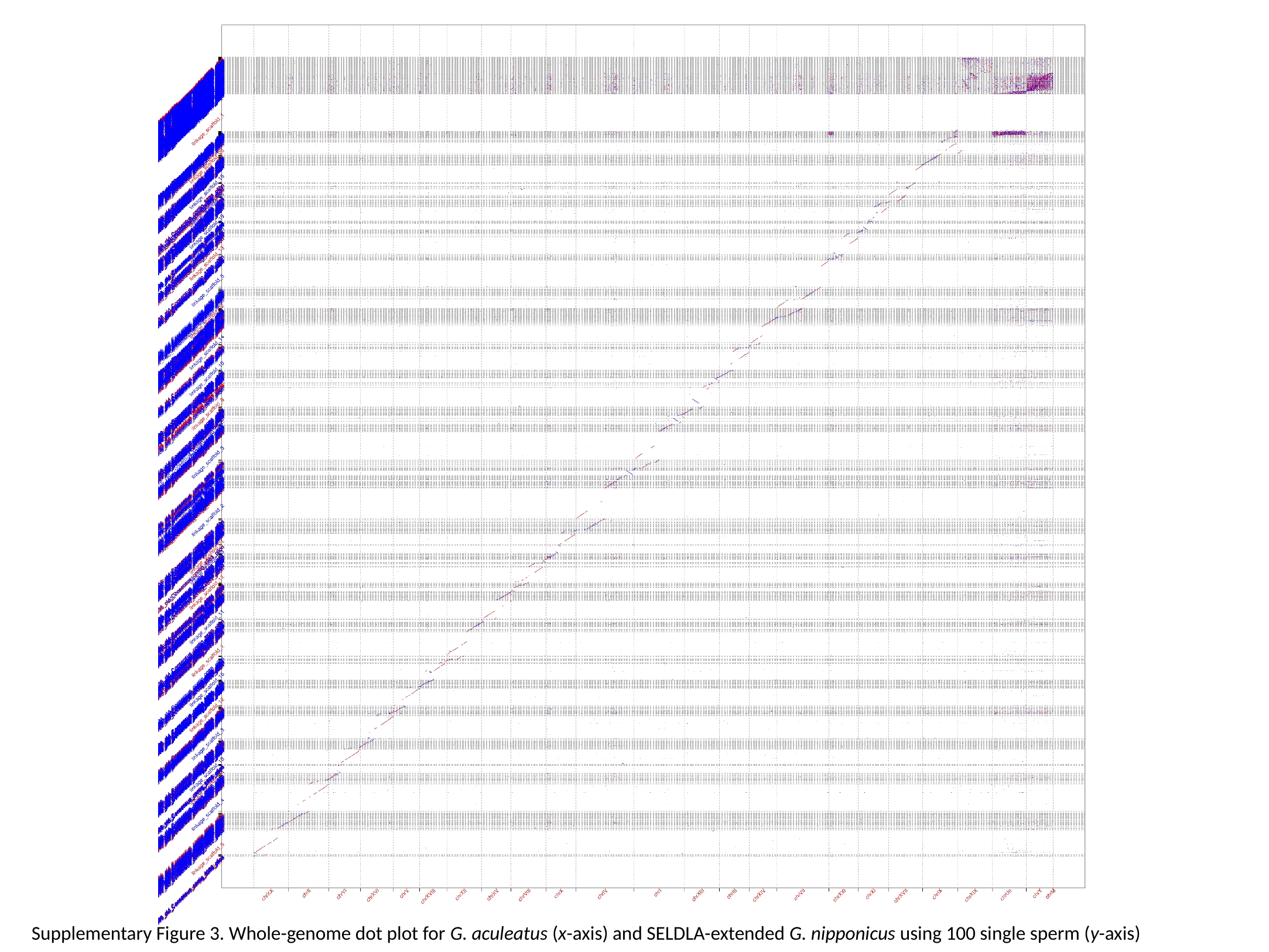

Supplementary Figure 3. Whole-genome dot plot for G. aculeatus (x-axis) and SELDLA-extended G. nipponicus using 100 single sperm (y-axis)

## Slide 4
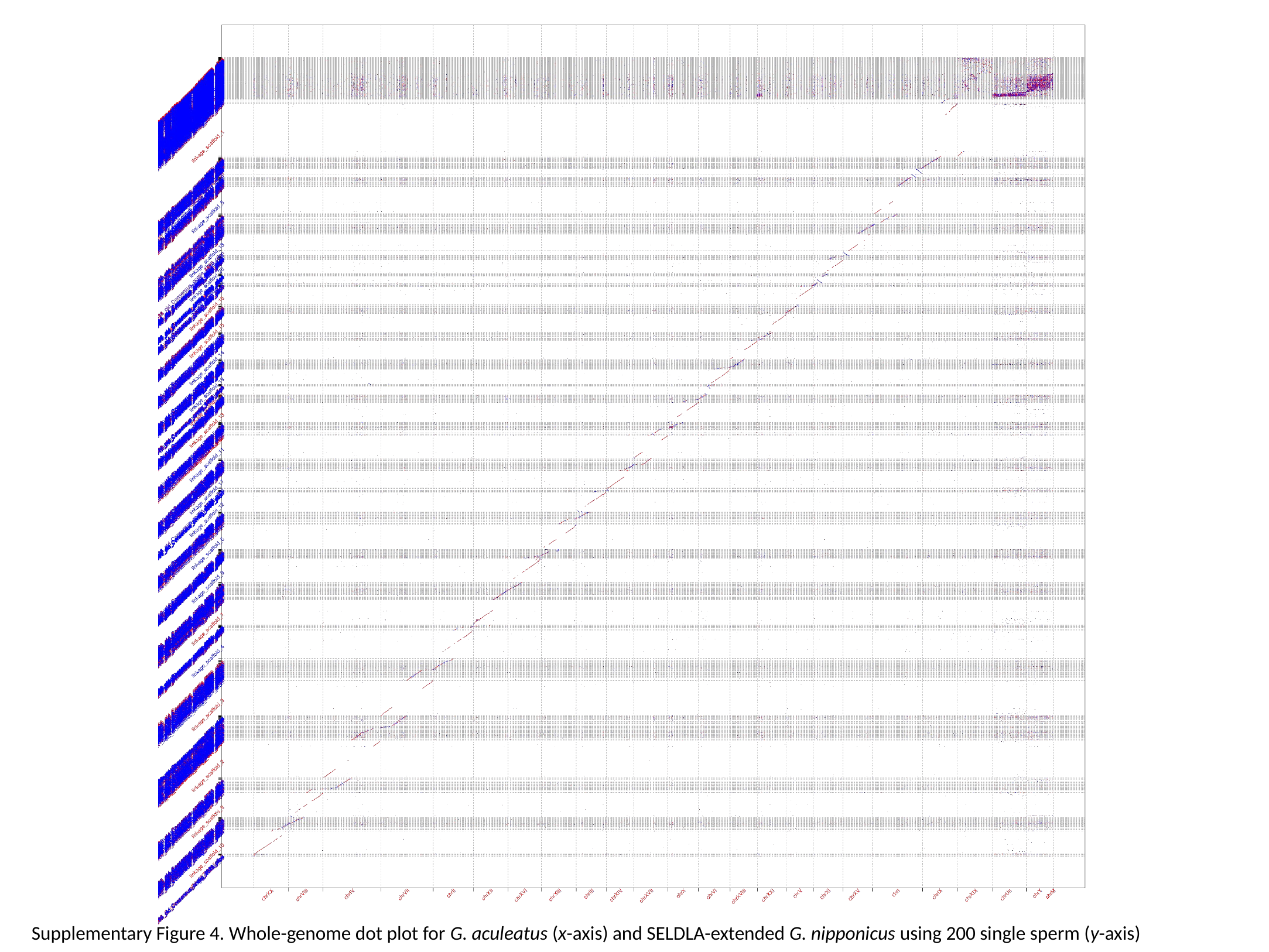

Supplementary Figure 4. Whole-genome dot plot for G. aculeatus (x-axis) and SELDLA-extended G. nipponicus using 200 single sperm (y-axis)

## Slide 5
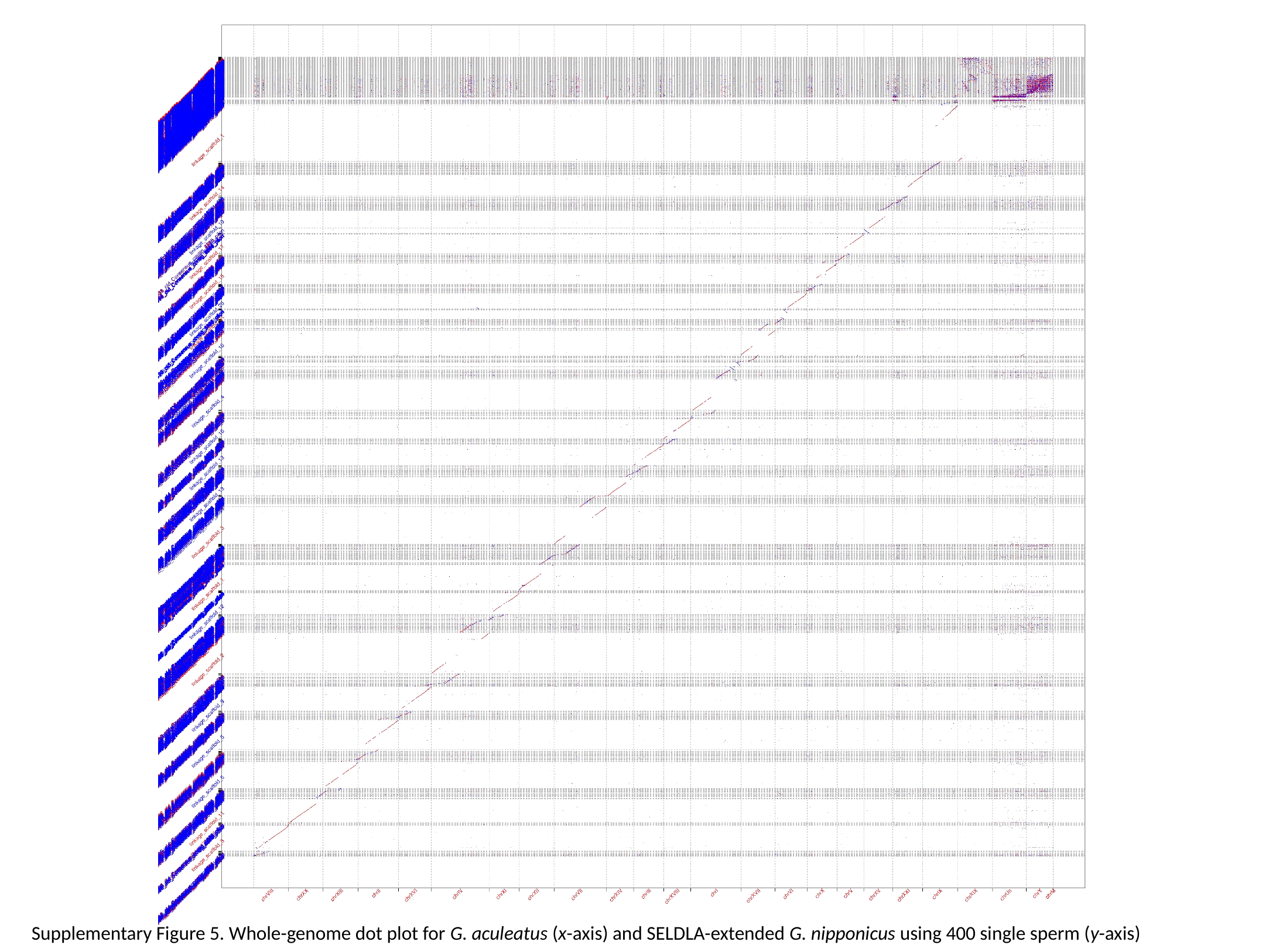

Supplementary Figure 5. Whole-genome dot plot for G. aculeatus (x-axis) and SELDLA-extended G. nipponicus using 400 single sperm (y-axis)

## Slide 6
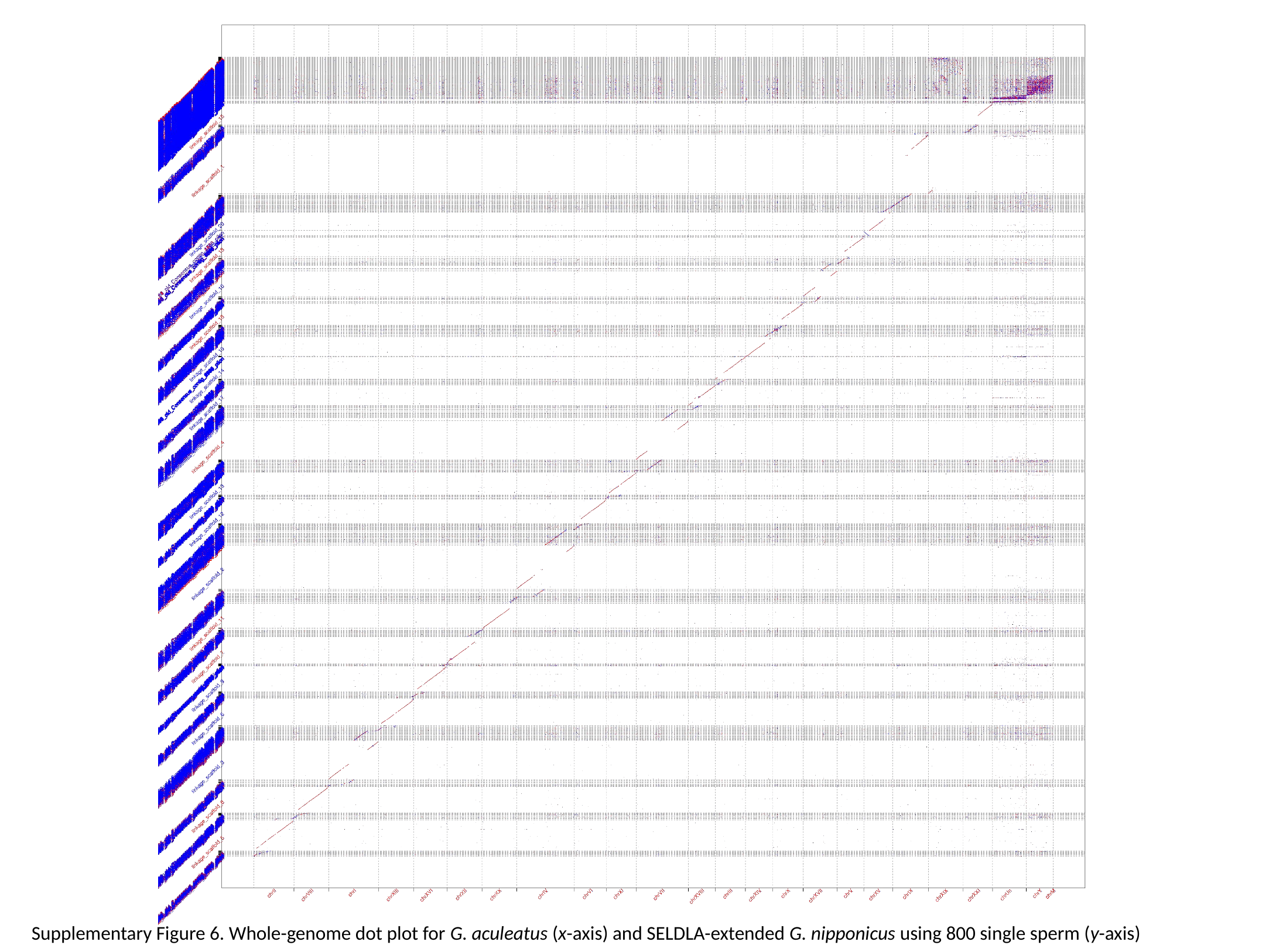

Supplementary Figure 6. Whole-genome dot plot for G. aculeatus (x-axis) and SELDLA-extended G. nipponicus using 800 single sperm (y-axis)
